# Application effect of catgut-embedding therapy combined with minimally invasive surgery for slow transit constipation: A protocol for systematic review and meta-analysis

**DOI:** 10.1097/MD.0000000000021875

**Published:** 2020-08-28

**Authors:** Yanpeng Xie, Yihua Fan, Wei Fan, Xiangdong Yang, Yanfei Xiang, Tianyu Zhao

**Affiliations:** a Hospital of Chengdu University of Traditional Chinese Medicine, Chengdu610072, Sichuan province; b First Teaching Hospital of Tianjin University of Traditional Chinese Medicine; c Tianjin University of Traditional Chinese Medicine, Tianjin; d Chengdu Anorectal Hospital, Chengdu, Sichuan Province, China.

**Keywords:** application effect, catgut-embedding therapy, meta-analysis, slow transit constipation, systematic review

## Abstract

**Background:**

Catgut-embedding therapy combined with minimally invasive surgery has been used for treating slow transit constipation (STC) widely. However, the application effect of catgut-embedding therapy combined with minimally invasive surgery for STC are unclear. This study aims to evaluate the application effect of catgut-embedding therapy combined with minimally invasive surgery for STC.

**Methods:**

Randomized controlled trials of catgut-embedding therapy combined with minimally invasive surgery in the treatment of STC will be searched in PubMed, EMbase, Cochrane Library, Web of Science, China National Knowledge Infrastructure, WanFang, the Chongqing VIP Chinese Science and Technology Periodical Database, and China biomedical literature database from inception to July, 2020. And Baidu Scholar, Google Scholar, International Clinical Trials Registry Platform, and Chinese Clinical Trials Registry will be searched to obtain more relevant studies comprehensively. Two researchers will perform data extraction and risk of bias assessment independently. Statistical analysis will be conducted in RevMan 5.3.

**Results:**

This study will summarize the present evidence by exploring the application effect of catgut-embedding therapy combined with minimally invasive surgery in the treatment of STC.

**Conclusions:**

The findings of the study will provide helpful evidence for the application effect of catgut-embedding therapy combined with minimally invasive surgery in the treatment of STC, facilitating clinical practice and further scientific studies.

**Ethics and dissemination:**

The private information from individuals will not publish. This systematic review also will not involve endangering participant rights. Ethical approval is not required. The results may be published in a peer-reviewed journal or disseminated in relevant conferences.

**OSF Registration number:**

DOI 10.17605/OSF.IO/7HVZB

## 1 Introduction

Constipation has become an important common disease affecting people's quality of life, which is prone to induce cardiovascular and cerebrovascular diseases.^[1]^ With the changes in social habits and dietary structure, the incidence of constipation has been increasing year by year in recent years. Some scholars have reported that the incidence of functional constipation in China is about 10% to 15%.^[2]^ Due to its unclear pathogenesis and unclear treatment direction, there is a lack of satisfactory therapeutic methods. According to the characteristics of intestinal and anorectal functions and dynamics that lead to constipation, there are three types: slow transit constipation (STC), outlet obstruction constipation and mixed constipation.

Colonic STC, also known as colonic weakness, is caused by dysfunction of large intestine and abnormal conduction, which leads to prolonged defecation cycle and difficulty in defecation.^[3]^ This concept was first put forward by Proton and Lennard-Joners in 1986. After nearly three decades of understanding, STC is now considered to be a type of refractory functional constipation characterized by slow delivery of feces in the intestinal tract, especially in women of childbearing age. Its main clinical features are less frequent defecation for a long time, once more than 3 days, or even 6 to 10 days 1 time, or no sense of defecation, dry and hard stool, defecation is laborious, and can also be combined with abdominal distension, anxiety, depression, sleep disorders and other symptoms, the symptoms are stubborn, and the treatment is intractable.^[4]^

In recent years, the incidence of chronic constipation has showed a slow upward trend and STC is the most common type of chronic constipation. The etiology is not clear. At present, the main treatment methods are stimulant, biofeedback and surgery. However, long-term use of laxatives can damage intestinal function, lead to drug dependence, more persistent constipation, and even cause melanosis of the colon. Surgical resection of the intestinal segment cannot be reduced, so careful selection should be made.^[5]^ The treatment of STC by traditional Chinese medicine, such as catgut-embedding therapy, has significant advantages, that is, significant curative effect, non-toxic side effects, high cure rate and no disease recurrence. Moreover, catgut-embedding therapy combined with minimally invasive surgery has less adverse reactions and better efficacy compared with normal surgery.^[6,7]^

In view of the current treatment status of constipation, safety and effectiveness become the main indicators to measure a new treatment. Because STC accounts for the majority of constipation, the previous treatment methods only solve one of them. Theoretically speaking, it cannot eliminate all the causes of defecation disorder, so it cannot achieve satisfactory curative effect. Catgut-embedding therapy mainly aims at slow transit problem, while anal minimally invasive surgery mainly solves outlet obstruction problem and achieves satisfactory curative effect. From total colectomy to subtotal colectomy, from peristaltic anastomosis with ileocecal preservation to retrograde peristalsis anastomosis, from single operation method to combined operation method, from open surgery to minimally invasive surgery. The establishment and development of surgical methods not only effectively improve the constipation symptoms of patients, but also reduce the pain and postoperative complications, the quality of life of patients was improved.^[8]^ However, there is no systematic review and meta-analysis regarding the application effect of catgut-embedding therapy combined with minimally invasive surgery for STC. Thus, this study will assess the application effect of catgut-embedding therapy combined with minimally invasive surgery for STC.

## 2 Methods

### 2.1 Study registration

This protocol of systematic review and meta-analysis has been drafted under the guidance of the preferred reporting items for systematic reviews and meta-analyses protocols (PRISMA-P). Moreover, it has been registered on open science framework (OSF) on July 20, 2020 (Registration number: DOI 10.17605/OSF.IO/7HVZB).

### 2.2 Ethics

Ethical approval is not required as there is no patient recruitment and personal information collection, and the data included in our study are from published literature.

### 2.3 Inclusion criteria for study selection

#### 2.3.1 Type of studies

Randomized controlled trials (RCTs) including catgut-embedding therapy combined with minimally invasive surgery for STC will be included. The language will be limited to Chinese and English.

#### 2.3.2 Type of participants

All the included cases conform to the “ RomeIVcriteria for Functional Gastrointestinal Disorders ”,^[9]^ regardless of nationality, race, age, gender, and source of cases.

#### 2.3.3 Type of interventions

The control group was treated with minimally invasive surgery only, and minimally invasive surgery type was not limited, such as partial amputation of internal anal sphincter, circumcision of rectal mucosa and repair of anterior / posterior process of rectum; the treatment group was treated with catgut-embedding therapy combined with minimally invasive surgery. The duration of treatment in both groups was not limited.

#### 2.3.4 Type of outcome measures

The main outcome measure was clinical efficacy. According to the score before and after treatment, ① Clinical cure rate: the proportion of patients who defecate more than 3 times a week. ② Clinical remission rate: the proportion of patients whose clinical symptoms did not reach the cure level but improved significantly compared with that before treatment. ③ Frequency of spontaneous defecation per week: the frequency of spontaneous defecation per week after treatment. Total effective rate = clinical cure rate + obvious efficiency + effective rate.^[10]^ ④ According to Bristol fecal characteristics score, there are seven grades in this score, which can be divided into seven grades, that is, 7 points for separated hard mass, 6 points for lumpy shape, 5 points for discontinuous and cracked sausage stool, 4 points for continuous and complete sausage stool, 3 points for soft lump, 2 points for mud like stool and 1 point for water sample.^[11]^⑤ Wexner constipation score: including defecation frequency, defecation difficulty, defecation time and other 8 items, the score range is 0 to 30 points, the higher the score is, the more severe the constipation degree is, and the score of healthy people is less than 8 points.^[12]^⑥ Gastrointestinal quality of life index (GIQLI): including 36 questions related to physiology, psychology, social activities and family life, the questionnaire objects were graded and scored within 2 weeks. The score range was 0 to 144 points. The higher the score, the better the gastrointestinal quality of life. The reference value of healthy people was (125.8 ± 13.0).^[13]^

### 2.4 Exclusion criteria

(1) The treatment group used other traditional Chinese medicine therapies, such as acupuncture and moxibustion, traditional Chinese medicine, and so on;
(2) The outcome indicators of the original study did not meet the requirements;
(3) As for duplicate published literature, select the literature with the most complete data;
(4) Literature with incorrect or incomplete research data that cannot be obtained after contacting the author.

### 2.5 Search strategy

PubMed, EMbase, Cochrane Library, Web of Science, China National Knowledge Infrastructure, WanFang, the Chongqing VIP Chinese Science and Technology Periodical Database, and China biomedical literature database were searched by computer to collect RCTs of catgut-embedding therapy combined with minimally invasive surgery in STC, and the retrieval time was from the establishment of each database to July 2020. At the same time, search Baidu, Google Scholar, International Clinical Trials Registry Platform, and Chinese Clinical Trials Registry to get more comprehensive data. Keywords were:“ catgut-embedding therapy”, “ minimally invasive surgery ”, “slow transit constipation”, et al. PubMed retrieval strategies are shown in Table 1.

**Table 1:**
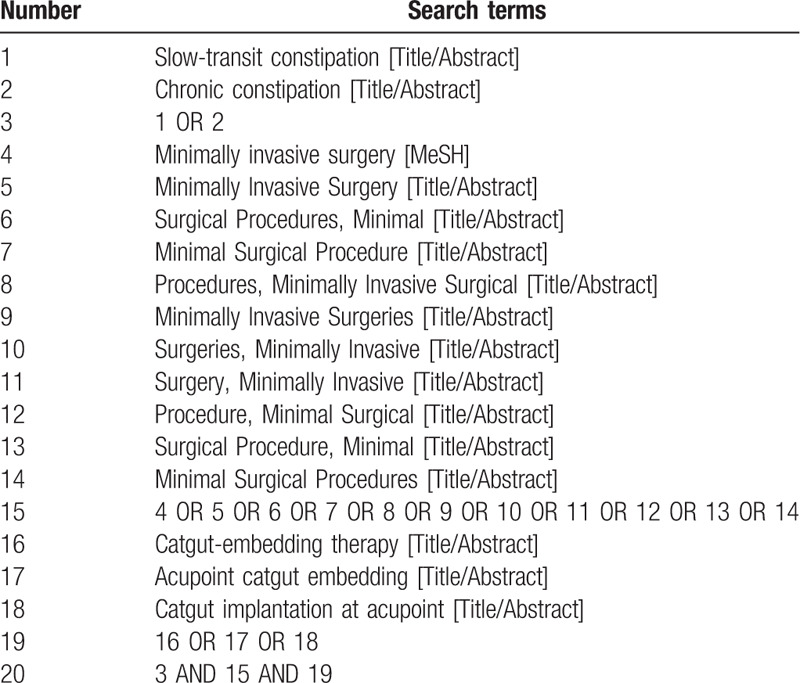
Search strategy in PubMed database.

### 2.6 Data extraction

Endnote X7 was used for literature management. Two researchers independently screened the literature, extracted the data and cross checked them. In case of disagreement, the third researcher was consulted to assist in judgment, and the lack of data was contacted with the author as much as possible to supplement. In the process of literature screening, first read the title and abstract, and then read the full text after excluding the obviously unrelated literature to determine whether it is included. Excel 2019 was used to set up a data extraction table to extract data.

**Figure 1:**
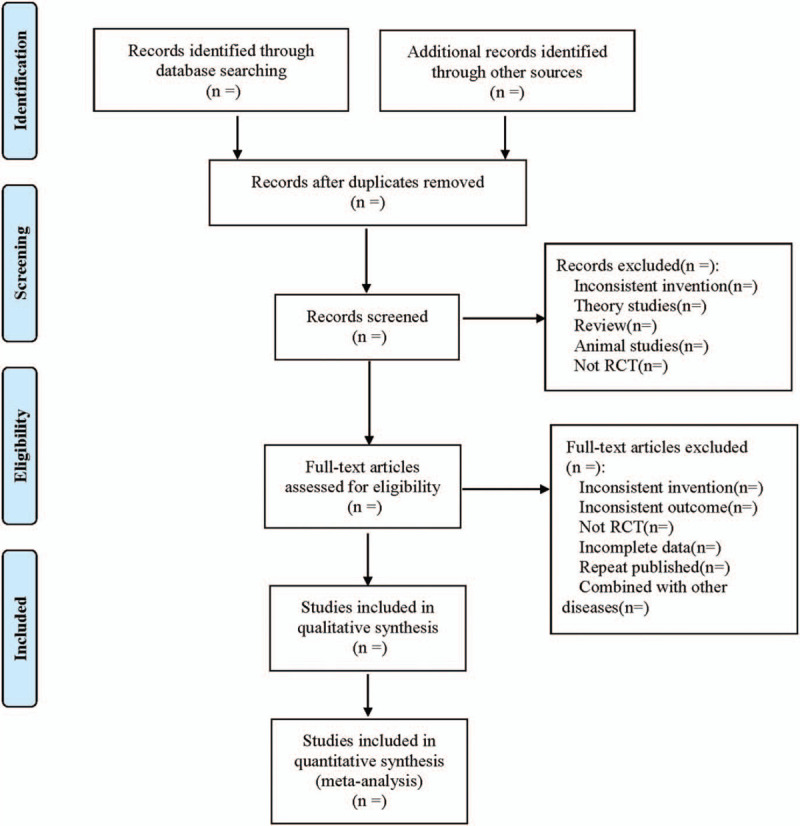
Flow diagram.

The extraction contents were as follows: ① Included basic research information (study title, first author, publication time, sample size, sex ratio, average age, etc); ② The treatment plan of the treatment group and the control group, such as the name and measure of the surgery used in the control group; the name and measure of the surgery, the acupoints, frequency, treatment course of catgut-embedding therapy used in the treatment group; ③ Risk evaluation items of bias in RCTs; ④ Related outcome indicators. The literature screening process is shown in Figure 1.

### 2.7 Risk of bias assessment

Two researchers independently evaluated the risk of bias in RCTs in accordance with the Cochrane Handbook of Systematic Reviewers, including the following items: random sequence generation, allocation concealment, blinding of participants and personnel, blinding of outcome assessment, incomplete outcome data, selective reporting, and other bias. The quality of studies was classified as being at of high, unclear or low risk of bias. In case of disagreement, a third researcher decided.

### 2.8 Statistical analysis

#### 2.8.1 Data synthesis

The RevMan 5.3 software provided by the Cochrane Collaboration was used for statistical analysis. ① For dichotomous variables, relative risk was used for statistics. For continuous variables, weighted mean difference was selected when the tools and units of measurement indicators are the same, Standardized Mean Difference was selected with different tools or units of measurement, and all the above were represented by effect value and 95% Confidence interval. ② Heterogeneity test: Q test was used to qualitatively determine inter-study heterogeneity. If *P*≥.1, there was no inter-study heterogeneity, If *P* < .1, it indicated inter-study heterogeneity. At the same time, *I*^*2*^ value was used to quantitatively evaluate the inter-study heterogeneity. If *I*^*2*^≤50%, the heterogeneity was considered to be good, and the fixed-effect model was adopted. If *I*^*2*^ > 50%,it was considered to have significant heterogeneity, the source of heterogeneity would be explored through subgroup analysis or sensitivity analysis. If there was no obvious clinical or methodological heterogeneity, it would be considered as statistical heterogeneity, and the random-effect model would be used for analysis.Descriptive analysis was used if there was significant clinical heterogeneity between the two groups and subgroup analysis was not available.

#### 2.8.2 Dealing with missing data

If data is missing or incomplete, we will contact the relevant authors to obtain the data. If not, this study will be removed.

#### 2.8.3 Heterogeneity and subgroup analysis

In order to reduce the clinical heterogeneity between studies, subgroup analysis was conducted according to the age, which were divided into minors, adults and the elderly. Subgroup analysis was carried out for the included studies according to the types of minimally invasive surgery, the time and acupoints of catgut-embedding therapy.

#### 2.8.4 Sensitivity analysis

In order to test the stability of meta-analysis results of indicators, a one-by-one elimination method will be adopted for sensitivity analysis.

#### 2.8.5 Reporting bias

For the major outcome indicators, if the included study was≥10, funnel plot was used to qualitatively detect publication bias. Egger's and Begg's test are used to quantitatively assess potential publication bias.

#### 2.8.6 Evidence quality evaluation

The Grading of Recommendations Assessment, Development, and Evaluation^[14]^ will be used to assess the quality of evidence. It contains 5 domains (bias risk, consistency, directness, precision, and publication bias). And the quality of evidence will be rated as high, moderate, low, and very low.

## 3 Discussion

STC is the most common type of functional constipation. It is known that the main cause is the colon dynamic disorder, the specific mechanism is not clear. Some researchers have studied fiber loss, autonomic neuropathy, and enteric and autonomic nervous systems. It is found that the number of Cajal cells in the colon of SCT patients decreased and the ultrastructure changed, the distribution was irregular and the cell volume decreased, and the cytoplasmic processes were interrupted or absent.^[15]^ Due to the unknown etiology and unclear pathogenesis, there is no effective treatment for it. Western medicine treatment includes drug treatment, surgical treatment, biofeedback therapy, etc., but long-term use of laxatives and prokinetic drugs will aggravate the damage of intestinal wall nerves. Surgical treatment belongs to traumatic treatment, which is easy to cause abdominal pain and flatulence and other side effects. Clinical study found that the effective rate of catgut-embedding therapy combined with surgical treatment in STC was improved.

Catgut-embedding therapy is a method of embedding catgut into acupoints or body, through which can stimulate the acupoints through the long-lasting and gentle physiological, physical and biochemical stimulation. By stimulating acupoints, the regulation of meridians and collaterals can be triggered so as to change the balance of endocrine and neurohumoral in human body. It is found that catgut-embedding at acupoints can improve the status of internal sphincter and pelvic floor muscle, reduce anal pressure and promote the recovery of constipation.^[16,17]^ It is considered that the continuous stimulation of acupoints with thicker catgut can enhance the tension and excitability of intestinal smooth muscle and promote intestinal peristalsis.^[18]^

The lack of accurate preoperative examination of the colon lesion leads to the diversification of STC surgical treatment. There is no perfect STC procedure. Surgical methods have experienced from simple to complex, then to individual selection, from a single surgical method to a variety of surgical methods, from transabdominal open surgery to laparoscopic minimally invasive surgery. To relieve the symptoms of constipation and reduce the incidence of complications is always the goal of STC surgical treatment. Compared with other surgery, minimally invasive surgery design has the advantages of good curative effect, small trauma, quick recovery and less complications. It can meet the individual needs of different constipation groups.^[19–21]^

However, there is no systematic review and meta-analysis assessing application effect of catgut-embedding therapy combined with minimally invasive surgery for treating STC. This is the first protocol for systematic review and meta-analysis evaluating the application effect of catgut-embedding therapy combined with minimally invasive surgery for treating STC. This systematic evaluation and meta-analysis can provide evidence-based evidence for clinicians to use catgut-embedding therapy and minimally invasive surgery in the treatment of STC. However, the study has some limitations. Due to different types of acupoints, the timing of minimally invasive surgery, the results were affected and the bias was caused. In addition, we only search for articles in Chinese and English, which may cause certain publication bias.

## Author contributions

**Data collection**: Yanpeng Xie and Yihua Fan

**Funding support**: Xiangdong Yang and Yanfei Xiang

**Literature retrieval**: Wei Fan and Tianyu Zhao

**Supervision**: Xiangdong Yang

**Writing – original draft**: Yanpeng Xie and Yihua Fan

**Writing – review & editing**: Xiangdong Yang and Yanfei Xiang
